# Swiss Small-Pox Statistics

**Published:** 1888-08-25

**Authors:** 


### Swiss Small-pox Statistics.

Mr. William Tebb, the well-known anti-vaccinator writes: "A paragraph in The Hospital to the effect that the recently-reported increase in the deaths from small-pox in Zurich is due to the repeal of the Vaccination Law in that canton does not, on examination, say much for the protective power of Jenner's infallible antidote. Small-pox, it is averred, had hitherto been prevented by compulsory vaccination, 'not a single case having occurred in 1882.' Allow me to observe that the cantonal law was repealed in 1883, and so transitory is the protection afforded by universal vaccination that before the year expires a variolous outbreak begins, and we are told that a more convincing demonstration of the fatal error of the anti-vaccination enthusiasts could scarcely be given! If the writer had extended his inquiries he would have learned that one of the severest outbreaks of small-pox of recent years in Switzerland occurred in Neuchatel, which is one of the three cantons where a compulsory law is still maintained, all the others, twenty in number, having repealed these oppresive enactments. The National Vaccination Law in Switzerland was abrogated on the 30th July, 1882, by a majority of 253,968, or about four to one, and the result has been so satisfactory that no attempt has been made by any section of the community to have it re-enacted."

*** It is possible to attach too much importance to the Zurich statistics, but Mr. Tebb's puzzle regarding them is easily solved. He says, in effect, "If compulsory vaccination were abolished in 1882, and small-pox began in 1883, does it not prove that the influence of the vaccination is very 'transitory?'" By parallel reasoning we might say, If vaccination has been almost abolished in Leicester since about 1878, does the continued absence of small-pox not prove the influence of the previous vaccinations to have been very permanent? Will Mr. Tebb here accept his own argument? Of course, he knows quite well that in Zurich many children have been born since 1882, on whom the abolished law did not bear, and that it is therefore not necessary to assume any transitory character of the previous vaccination to explain the outbreak. Indeed, so far as it goes, the Zurich experience should be a warning to Leicester. We know nothing of the outbreak in Neuchatel, but in epidemics in this country the rule is that the risks, both of attack and of death, are much greater among the unvaccinated than among the vaccinated; and, for anything Mr. Tebb advances to the contrary, it may have been such an experience in Neuchatel that has taught the people the need for retaining their law. He alleges that a Boston doctor has been killed by vaccination. Granting what Mr. Tebb here assumes, that the blood poisoning in this case was really due to vaccination, what is the inference? That vaccination should be abolished? If so, should railway travelling be abolished because some "well-known" personage once got killed on a railway? Or should compulsory education be abolished because some child has died under it? Or should women be allowed to go about the streets naked because a lady's dress occasionally causes her death?—Ed. *T. H.*

